# Artemisinin pressure in field isolates can select highly resistant *Plasmodium falciparum* parasites with unconventional phenotype and no K13 mutation

**DOI:** 10.1128/aac.01541-24

**Published:** 2025-02-04

**Authors:** Lucie Paloque, Luana Mathieu, Marion Laurent, Romain Coppée, Stéphanie Blandin, Pascal Campagne, Jean-Michel Augereau, Lise Musset, Françoise Benoit-Vical

**Affiliations:** 1 LCC-CNRS, Laboratoire de Chimie de Coordination, Université de Toulouse, CNRS 137668 , Toulouse, France; 2 MAAP, New antimalarial molecules and pharmacological approaches, Inserm ERL 1289, Toulouse, France; 3 Institut de Pharmacologie et de Biologie Structurale (IPBS), Université de Toulouse, CNRS, Université Toulouse III – Paul Sabatier 137668 , Toulouse, France; 4 Laboratoire de Parasitologie, Centre National de Référence du Paludisme, Pôle Zones Endémiques, WHO Collaborating Center for Surveillance of Antimalarial Drug Resistance, Institut Pasteur de la Guyane 638330 , Cayenne, French Guiana; 5 Laboratoire de Parasitologie-Mycologie, Université de Rouen Normandie 27040 , Mont-Saint-Aignan, Normandy, France; 6 CNRS, Inserm, UPR9022/U1257, Mosquito Immune Responses (MIR), Université de Strasbourg 27083 , Strasbourg, France; 7 Institut Pasteur, Bioinformatics and Biostatistics Hub, Université Paris Cité 555089 , Paris, France; The Children's Hospital of Philadelphia, Philadelphia, Pennsylvania, USA

**Keywords:** malaria, *in vitro *selection, artemisinin resistance, non-K13 polymorphism

## Abstract

Artemisinin resistance, which poses a serious threat to malaria control efforts, is monitored in the field by delayed parasite clearance in patients, elevated parasite survival rate in the ring-stage survival assay, and mutations in the *Plasmodium falciparum kelch13* gene. However, sporadic cases of artemisinin-resistant malaria do not meet all of these criteria, highlighting that our understanding of artemisinin resistance is still incomplete. Here, we selected for artemisinin resistance *in vitro* in nine *P. falciparum* field isolates from Africa, Asia, and South America. Artemisinin pressure selected highly resistant parasites in all lineages, but only one acquired a validated *pfkelch13* mutation. Phenotypic tests evidenced an unconventional response of selected parasites that can be artemisinin-resistant only at an advanced ring age, making them undetectable by the standard “0–3 h” test used to monitor resistance. These results highlight the need for broader approaches to artemisinin resistance monitoring.

## INTRODUCTION

Resistance of the malaria parasite *Plasmodium falciparum* to artemisinin (ART), the core component of the frontline antimalarial artemisinin-based combination therapies (ACTs), is clear and presents a threat to malaria control and elimination (1), particularly in Africa, which is facing *de novo* emergence and/or spread of such resistance (2, 3). ART resistance is associated with a delayed parasite clearance half-life (PCt_½_ >5 h) in patients following ART-based treatment (1, 4) and is characterized by a greater ability of young parasites to survive ART exposure (5), as has been confirmed *ex vivo* and *in vitro* (6, 7). Therefore, the ring-stage survival assay (RSA^0–3h^), based on a brief exposure of 0–3 hold rings to a pharmacologically relevant dose of dihydroartemisinin (DHA), has become the reference phenotypic test for assessing ART resistance (8, 9). At the molecular level, mutations in the *P. falciparum kelch13* (*pfk13*) gene have been confirmed as a major driver of ART resistance (1, 10–12). Mutations in the *pfcoronin* gene also confer resistance to ART but have only been selected *in vitro* and have not yet been observed in field-resistant isolates (13). Interestingly, several field isolates displaying delayed parasite clearance (14, 15) and/or increased survival rates in the RSA^0–3h^ test (16–19) did not carry *pfk13* mutations, underlining the need for a better understanding of ART resistance. For this purpose, and to improve the surveillance of resistance emergence, we explored here the selection process for ART resistance. In a previous study using African *P. falciparum* isolates (collected in Mali 5 years after the introduction of ACTs in the region), we showed that ART-susceptible parasites can differ significantly in their ability to recover after DHA exposure (20). We classified these parasites as having slow or fast recovery phenotypes. We hypothesized that such phenotypic differences could be the first signs of selection for ART resistance. To investigate this, we chose nine (originally ART-susceptible) parasite field isolates from different geographical origins (West Africa, Southeast Asia, and South America) to study *in vitro* ART resistance selection (using two different protocols for two of them) over a 3-year period.

## MATERIALS AND METHODS

### *P. falciparum* lines and culture

The *P. falciparum* lines used were (i) KMT001, KMT004, KMT012, KMT102, and SMT010 (all ART-susceptible) collected in Mali (20); (ii) IPC5188 (ART-susceptible) and IPC8262 (ART-resistant K13-C580Y) collected in Cambodia; (iii) O141-A, S691, and U236 (all ART-susceptible) collected in French Guiana; and (iv) the laboratory lines F32-ART (ART-resistant K13-M476I) and F32-TEM (ART-susceptible) (7). *P. falciparum* lines were cultured as previously reported (11) in human type O red blood cells (French Blood Bank, EFS) in RMPI-1640 medium (Dutscher) supplemented with 5% to 10% human serum (EFS), 0.055% hypoxanthine, 0.55% Albumax II (Fisher Scientific), 1 mM L-glutamine, and 11 µg/mL gentamicin, at 37°C with 5% O_2_, 5% CO_2_, and 90% N_2_, except for lines F32-ART and F32-TEM, which were grown in human type O red blood cells in RMPI-1640 medium supplemented with 5% human serum, at 37°C with 5% CO_2_.

### Selection for artemisinin resistance

ART resistance was selected by sequential parasite exposure to increasing doses of ART (from 10 nM to 15 µM for 24 h) using the protocol that allowed to select the first ART-resistant laboratory line F32-ART (7, 10) or a constant dose of DHA (for 6 h at 700 nM, corresponding to the peak plasma concentration in patients [8, 9]). Briefly, 0–24 h ring-stage parasites at 3% parasitemia and 3% hematocrit in culture medium supplemented with 10% human serum were exposed to either ART or DHA. The infected red blood cells were then washed once with RPMI-1640 medium and replaced in a new flask with a drug-free culture medium (at 10% human serum). Parasitemia was monitored microscopically by Diff-Quick-stained thin blood smears until the culture reached 1% parasitemia (defined here as the time to recovery). The parasite culture was then transferred to normal culture conditions (5% human serum) until the next drug pressure cycle. The average duration of “off-drug culture” between two cycles of drug pressure was 20 days. One to three drug pressure cycles were performed with ART doses ranging from 10 to 750 nM, and then three drug pressure cycles were performed at each dose from the 1 to 15 µM dose. The ART concentrations used for each drug pressure cycle are listed in Table S1.

### Evaluation of parasite susceptibility to ART and DHA

Parasite susceptibility to ART and DHA was assessed by IC_50_ determination using the SYBR Green I (21) or tritiated hypoxanthine incorporation methods (22), the ring-stage survival assay (9), and the ring-stage kinetic assay (RSKA), which was designed to determine the recrudescence kinetics of parasites over 7 days after brief exposure to DHA (20). For all experiments, parasites were synchronized either at the 0–24 h post-invasion ring stage by 5% D-sorbitol treatment or by Percoll-sorbitol treatment to obtain tightly synchronized ring-stage parasites. To do this, schizonts were first harvested on Percoll 76% solution and re-cultured with fresh red blood cells. Three, 4, or 8 h later (depending on the required synchronization window), the parasites were treated with 5% D-sorbitol, resulting in 0–3 h, 0–4 h, or 0–8 h old ring-stage parasites, respectively. In the RSA, ring-stage parasites at 0.5% parasitemia, 2% hematocrit, and 10% human serum were exposed in duplicate in 48-well plates to 700 nM DHA or 0.1% DMSO for 6 h (final volume of 1 mL), then washed once in 10 mL RPMI-1640 medium, and returned to drug-free culture conditions (10% human serum) for the next 66 h. Parasitemia in each well was calculated on stained thin blood smears by counting twice (by two independent experienced microscopists) 2,500 red blood cells per control condition and 10,000 red blood cells per treated condition at least. In the RSKA, ring-stage parasites at 3% parasitemia, 2% hematocrit, and 10% human serum were exposed to 700 nM DHA for 6 h in 6-well plates (final volume of 5 mL), then washed once in 40 mL RPMI-1640, and returned to drug-free culture conditions (at 10% human serum) for the next 7 days. The culture medium was changed on days 3 and 5. Parasitemia was monitored from day 3 to day 7 by counting 5,000 red blood cells per condition on stained thin blood smears. RSA and RSKA values depend on the parasite’s abilities to survive DHA treatment and the parasite’s growth rates but also on blood batch and other environmental changes.

### Kinetics of the intraerythrocytic development cycle

Parasites from the selected and corresponding parental lines were tightly synchronized by Percoll (T 0 h)/sorbitol (T 4 h) treatment at 0–4 h post-invasion, adjusted to 1% parasitemia and 2% hematocrit, and placed in normal culture conditions. Parasites were then harvested, and blood smears were made at 4, 8, 24, 28, 32, 48, 52, 56, and 60 h. The percentage of each stage was calculated by counting twice (by two independent experienced microscopists) 250 parasites per condition. Morphologically, ring-stage parasites were defined as parasites with a ring shape and uncolored cytoplasm, trophozoite stages as parasites with colored cytoplasm, and schizonts as parasites with punctate nuclear coloration of the developing merozoites.

### Competition experiments

The competitive advantage of the selected lines over their parental lines was evaluated after 2 months of coculture. Six parasite cultures were prepared: one for each parental line (KMT001, KMT012) and their corresponding selected lines (KMT001p27/DHA, KMT012p51/ART), one culture mixing KMT001 with KMT001p27/DHA, and one culture mixing KMT012 with KMT012p51/ART. On day 0, the parasitemia of each culture in drug-free culture medium was adjusted to 1% (0.5% of each line for mixed cultures) with 0–24 h ring-stage parasites, at 2% hematocrit and 5% human serum. Parasite cultures were maintained in parallel under drug-free conditions with the same batches of medium, blood, and serum for 2 months by changing the medium and adjusting the parasitemia to 1, 1, and 0.5% every Monday, Wednesday, and Friday, respectively. On day 0 and day 60, the phenotype of the different parasite cultures was assessed for ART susceptibility using the RSKA^0–24h^.

### Generation of gametocytes and mosquito infection

*P. falciparum* gametocytes were generated *in vitro* using similar procedures as previously described (23). All cultures were maintained in human red blood cells (EFS) at 3% hematocrit and in complete medium (RPMI-1640 with L-glutamine and 25 mM HEPES supplemented with 10% human serum [EFS] and 10 mM hypoxanthine [c-c-Pro, Oberdorla]) at 37°C under a 5% O_2_, 5% CO_2_ and 90% N_2_ atmosphere. Asexual parasite cultures were maintained at a maximum parasitemia of 3%. Gametocyte cultures were seeded in a 6-well plate at 0.5% parasitemia and maintained in culture with daily medium changes for 16 days prior to infection. The quality and density of gametocytes were regularly assessed on blood smears. *Anopheles stephensi* mosquitoes were bred using classical procedures with the approval of the French Ministère de l’Enseignement Supérieur et de la Recherche under the reference APAFIS #20562-2019050313288887v3. All developmental stages were maintained at 27°C and 75% humidity in a 12 h/12 h day/night cycle. Gametocyte-infected erythrocytes were mixed with uninfected erythrocytes and human serum (1.5 million gametocytes stage V/mL reconstituted blood, 50% hematocrit) and offered to starved *A. stephensi* female mosquitoes using a Hemotek system mounted with parafilm to maintain the blood meal at 37°C. Unfed females were discarded 3–4 h after feeding, and the remainder were maintained at 27°C, 70% humidity for 7–8 days before dissection. Midguts were dissected in PBS, stained in 0.2% mercurochrome in water for 6 min at room temperature, and then rinsed at least three times in PBS for 5–10 min each before mounting and imaging on a Zeiss Axio Zoom.v16 microscope.

### Single-nucleotide polymorphism genotyping

Parasite DNA was extracted from red blood cell pellets using the High Pure PCR Template Preparation Kit (Roche Diagnostic). All PCR experiments were performed using the DreamTaq Hot Start DNA Polymerase (Fisher Scientific). Sanger sequencing was performed by the GenoScreen company (Lille, France). All primer sets used are listed in Table S2.

## RESULTS

### Genotypes of parental and selected parasites

**Fig 1:**
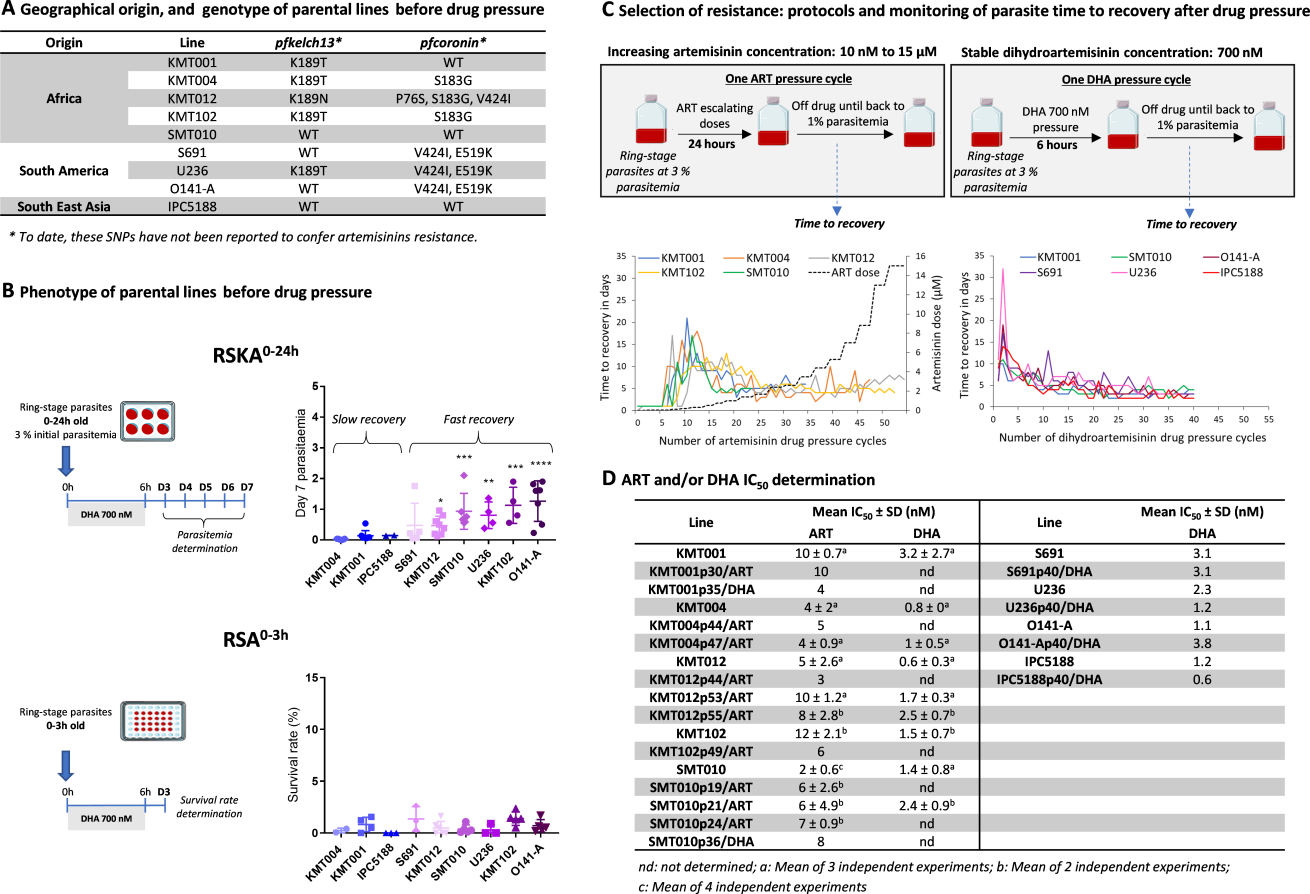
Overview of the nine parental lines, drug pressure protocols, and parasite monitoring during the selection process. (**A**) Geographical origin and genotype of the parental lines before drug pressure. Genotyping of the *pfk13* and *pfcoronin* genes in the parental lines was performed by Sanger sequencing; WT: wild type. (**B**) *In vitro* evaluation of the susceptibility of the parental lines to DHA exposure (700 nM for 6 h) in RSKA^0–24h^ and RSA^0–3h^ (mean ± SEM) before starting the drug resistance selection process. Statistical significance (one-way ANOVA with Dunnett’s *t*-test) was determined for each line compared to KMT004 (used here as a reference). ^*^P value <0.05, ^**^P value <0.01, ^***^P value <0.001, ^****^P value <0.0001. The RSKA^0–24h^ values of the African lines include those previously published.^19^ (**C**) *In vitro* selection of ART-resistant lines by exposing the parental lines to increasing doses of ART ranging from 10 nM to 15 µM or a fixed dose of DHA (700 nM). For each drug pressure cycle, post sorbitol ring-stage parasites at 3% parasitemia were exposed to the drug for 24 h (ART pressure) or 6 h (DHA pressure). After drug withdrawal, the parasites were returned to drug-free culture conditions until the next cycle. Time to recovery of the different lines after each pressure cycle, defined as the time to reach 1% parasitemia. (**D**) Evaluation of chemosensitivity to ART and DHA (using the SYBR Green I or the tritiated hypoxanthine incorporation methods) of the *P. falciparum* parental lines used in this study and of the selected lines annotated pX (“p” for pressure, “X” for the number of pressure cycles and ART/DHA for the drug used for pressure).

In the nine parental lines, the absence of known molecular markers associated with ART resistance was confirmed by Sanger sequencing of the *pfk13* and *pfcoronin* genes. Part of the parental lines carried the K13 mutations K189T/N and/or the coronin mutations P76S, S183G, V424I, and E519K (Fig. 1A), which have never been associated with ART resistance (1). During the drug pressure experiments, only the SMT010/ART lineage acquired an additional mutation in the *pfk13* gene, i.e*.,* P413A mutation, between the 15th and 18th drug pressure cycle (Table S3). Note that the ongoing whole-genome analysis has not identified any variants in *pfubp1* and *pfap-2µ* genes also associated *in vitro* with ART resistance (24).

### Phenotypes of selected parasites

After adaptation to *in vitro* culture, phenotypes of the nine parental lines were determined in both RSKA^0–24h^ and RSA^0–3h^. Prior to selection for ART resistance, three lines exhibited slow recovery phenotypes, and six lines exhibited fast recovery phenotypes according to RSKA^0–24h^ (Fig. 1B) (20), while all of these parasite lines exhibited low survival rates according to RSA^0–3h^ (Fig. 1B). Five parasite lines (KMT001, KMT004, KMT012, KMT102, and SMT010) from Mali (Africa) were exposed to intermittent and increasing concentrations of ART (from 10 nM to 15 µM for 24 h; Table S1). In parallel, the KMT001 and SMT010 lines were also exposed to intermittent but fixed doses of DHA (700 nM for 6 h), as were the S691, U236, O141-A (from French Guiana, South America) and IPC5188 (from Cambodia, Southeast Asia) lines (Fig. 1C). Monitoring the recovery time of parasite cultures after each cycle of drug pressure provided the first evidence of parasite adaptation to ART or DHA exposure over time, including a decrease in recovery time for the eleven selected parasite lineages (Fig. 1C) (here, lineage refers to all descendants of a parental isolate, while line refers to the parasites obtained at a specific time point in the selection process). No shift in ART and/or DHA IC_50_ values was observed even after numerous drug pressure cycles (Fig. 1D).

**Fig 2:**
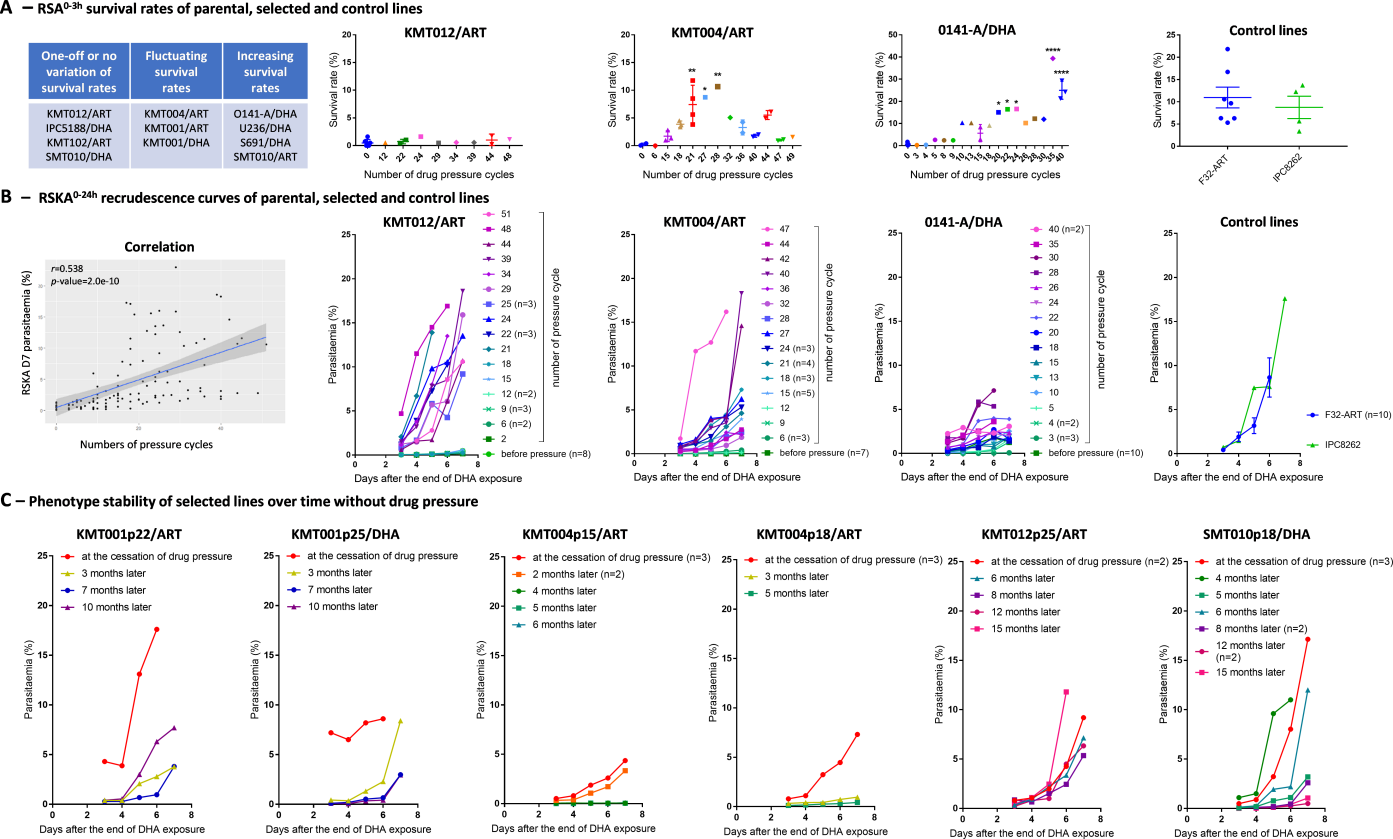
*In vitro* evaluation of the sensitivity of the main representative parasite lines to DHA throughout the drug resistance selection process using the RSA^0–3h^ and RSKA^0–24h^ methods. (**A**) RSA^0–3h^ mean survival rate (±SEM) of parasites exposed to 700 nM DHA for 6 h compared to the control condition for each lineage at different times of the selection process (after X-drug cycles) and for the F32-ART laboratory strain (PfK13-mutated M476I) and the Cambodian field isolate IPC8262 (PfK13-mutated C580Y). Statistical significance (one-way ANOVA with Dunnett’s *t*-test) was determined for each pressure cycle compared to p0. ^*^P value <0.05, ^**^P value <0.01, ^****^P value <0.0001. (**B**) Correlation between parasitemia on day 7 in the RSKA^0–24h^ and pressure cycle for all selected lineages. Recrudescence curves of parasites over 7 days (parasitemia as a function of time) after the end of DHA exposure for each lineage at different times of the selection process (after X drug cycles) and for the control lines F32-ART and IPC8262. Some curves end earlier because high parasitemia leads to an impaired ability of parasites to reinvade red blood cells. (**C**) Recrudescence curves of parasites over 7 days (parasitemia as a function of time) after the end of DHA exposure (700 nM for 6 h) in the RSKA^0–24h^ for each line at different times after the end of the selection process.

Different evolutionary profiles of RSA^0–3h^ survival rates across pressure cycles were observed depending on the parasite lineage: four lineages presented one-off or no variation in survival rates (as for the KMT012/ART lineage), three lineages presented fluctuating survival rates (as for the KMT004/ART lineage), and four lineages presented increasing survival rates (as for the O141-A/DHA lineage) (Fig. 2A; Fig. S1). No growth defect was observed in the drug-selected lines, with the average growth rate in the RSA mock-treated samples ranging from 5 to 13 depending on the lineage (File S1). Fluctuating survival rates may reflect the selection of resistant clones at one point of the selection process but associated with a fitness cost that may be responsible for their disappearance from the bulk culture during the “off-drug” periods.

In parallel, based on the RSKA^0–24h^ results, we observed that the capacity for parasite recrudescence increased with the number of drug pressure cycles applied in all lineages, as evidenced by parasitemia on day 7 (Fig. 2B; Fig. S2) or by the slope of recrudescence curves (Fig. S3), both of which correlated positively with the number of drug pressure cycles applied (*r* = 0.538 and *r* = 0.564, respectively, both with *P* <0.0001). The increased parasitemia on day 7 according to RSKA^0–24h^ was similar to that of the ART-resistant reference strain F32-ART (*pfk13* M476I mutated) and the Cambodian field isolate IPC8262 (*pfk13* C580Y mutated) (Fig. 2A and B). The ART resistance phenotype of selected lines was still observed whatever the exposure duration to DHA (from 6 to 72 h) or doses of DHA (from 700 nM to 7 µM) (Fig.S4).

### Stability of the selected phenotypes

The stability over time (in the absence of drug pressure) of the selected lines regarding their ability to recover after DHA exposure was also evaluated in six different lines after stopping the drug pressure experiments at different times. Three lines, KMT004p15/ART (“p” for number of pressure cycles), KMT004p18/ART, and SMT010p18/DHA (with parasitemia on day 7 ranging from 4% to 17% in RSKA^0–24h^), completely lost their ability to recover after DHA exposure (becoming phenotypically ART-susceptible again) in 4, 5, and 12 months of drug-free culture, respectively. The lines KMT001p25/DHA and KMT001p22/ART, with a parasitemia of 8% and 18%, respectively, on day 7, were associated with a clear but partial decline of their RSKA^0–24h^ values even after 10 months of drug-free culture. In contrast, the recrudescence capacities of the KMT012p25/ART line remained stable over 15 months (Fig. 2C).

### The parasitic “age of resistance” depends on the parasite line

**Fig 3:**
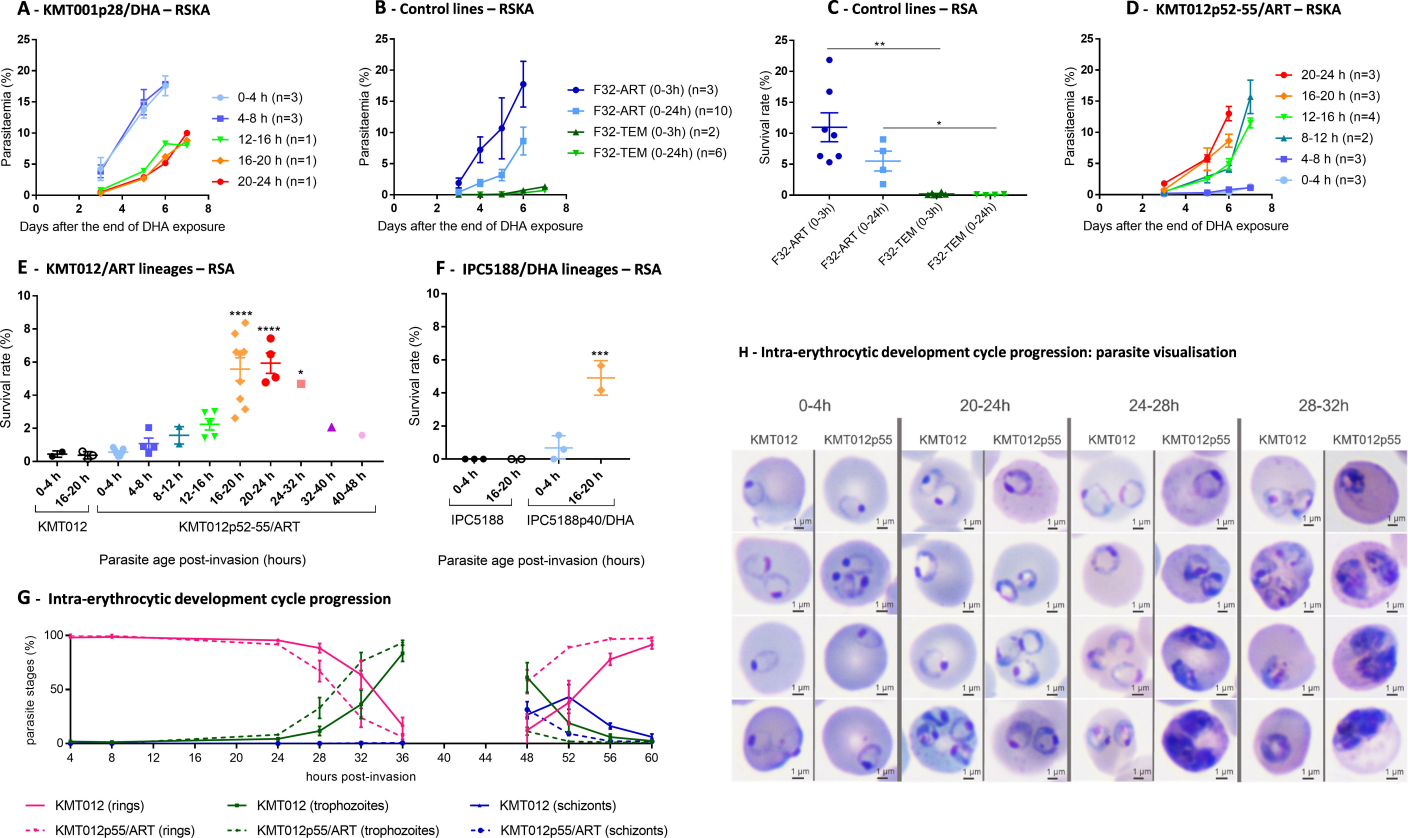
*In vitro* evaluation of parasite sensitivity to DHA exposure (700 nM for 6 h) as a function of post-invasion age. (**A**) Recrudescence curve (RSKA) (±SEM) of parasites over 7 days after the end of DHA exposure for the KMT001p28/DHA line according to their post-invasion age at the time of drug exposure. (**B and C**) *In vitro* evaluation of the sensitivity to DHA exposure (700 nM for 6 h) in the RSKA (mean parasitemia ± SEM) and in the RSA (mean survival rate ± SEM) according to their post-invasion age for the F32-ART (artemisinin-resistant) and F32-TEM (artemisinin-susceptible) strains. Statistical significance was determined by unpaired *t*-test, Mann-Whitney. ^*^P value <0.05, ^**^P value <0.01. (**D**) Recrudescence curve (±SEM) of parasites over 7 days after the end of DHA exposure for the KMT012p52-55/ART lines according to their post-invasion age at the time of drug exposure. (**E and F**) RSA mean survival rate (±SEM) of parasites according to their post-invasion age at the time of DHA exposure for KMT012p52-55/ART, IPC5188p40, and their corresponding parental lines KMT012 and IPC5188. Statistical significance (one-way ANOVA with Dunnett’s *t*-test) was determined for each parasite age compared to 0–4 h (selected line). ^*^P value <0.1, ^***^P value <0.001, ^****^P value <0.0001. (**G and H**) Relative proportion of each developmental stage over the life cycle in tightly synchronized cultures of the parental line KMT012 and the selected line KMT012p55/ART. Values are based on three independent experiments. The percentage of each stage was counted by two independent microscopists for at least 500 parasites in total per condition. Morphologically, ring-stage parasites are defined as parasites with a ring shape and uncolored cytoplasm, trophozoite stages are defined as parasites with colored cytoplasm, and schizonts are defined as parasites with punctate nuclear staining of the forming merozoites. Microscopic examination of KMT012 and KMT012p55/ART parasite across the intraerythrocytic development cycle by (DiffQuick-stained blood smear, magnification 100 with immersion oil, scale bar: 1 µm).

The increase in parasite recrudescence capacity (detected by RSKA^0–24h^) was not systematically visible on day 3 (i.e*.,* the time of survival rate monitoring according to RSA^0–3h^), which may explain the discrepancy in parasite survival sometimes observed between the two assays. Another explanation could be the different sensitivity to ART depending on the age of the ring-stage parasites when exposed to DHA (0–3 h in RSA and 0–24 h in RSKA). When RSKA was performed using a line with a classic resistance profile (with an elevated survival rate according to RSA^0–3h^), the recrudescence capacities were greater for young rings (0 to 8 h old) than for older rings (Fig. 3A), as was also observed in the ART-resistant reference strain F32-ART (Fig. 3B and C). In contrast, the oldest parasites (20–24 h old) of the KMT012p52-55/ART lines had a greater capacity for recrudescence, with parasitemia >12% on day 6, than younger parasites (0–8 h old), with parasitemia <2% on day 7 (Fig. 3D). The results of RSA performed under the same conditions were consistent with the survival rates (<1% for 0–4 h old parasites), which gradually increased as the parasite cell cycle progressed, reaching a maximum of 6% for 20–24 h old parasites (Fig. 3E). Interestingly, when the survival rate shifted from only 1.1% for 4–8 h rings to 1.6% for 8–12 h rings, the corresponding parasitemia on day 7 according to RSKA increased from 1.1% to 15.7%, suggesting that RSKA may be more informative than RSA regarding parasite sensitivity to DHA. The ability of selected parasites to survive DHA exposure only at the old ring stage was also observed in the Asian IPC5188p40/DHA line (Fig. 3F), indicating that this particular phenotype is not restricted to one specific genetic background. The survival rates according to RSA of both parental lines were less than 1% regardless of whether the ring-stage parasites were 0–4 h or 16–20 h old at the time of DHA exposure (Fig. 3E and F). For the KMT012/ART lineage, we examined the intraerythrocytic development cycle profile of both the parental and selected lines (starting with tightly synchronized parasites at 0–4 h post-invasion) under drug-free conditions by monitoring the proportion of parasites in each stage at different times. The selected line had a faster IDC than the parental line, with a mean difference of 3 h for the transition from rings to trophozoites (50% of each stage) (Fig. 3G and H; Fig. S5). Thus, the ability of the KMT012p55/ART line to survive DHA exposure at the old ring stage was not associated with a prolonged ring stage.

### Competitive advantage and ability of selected parasites to infect mosquito vectors

**Fig 4:**
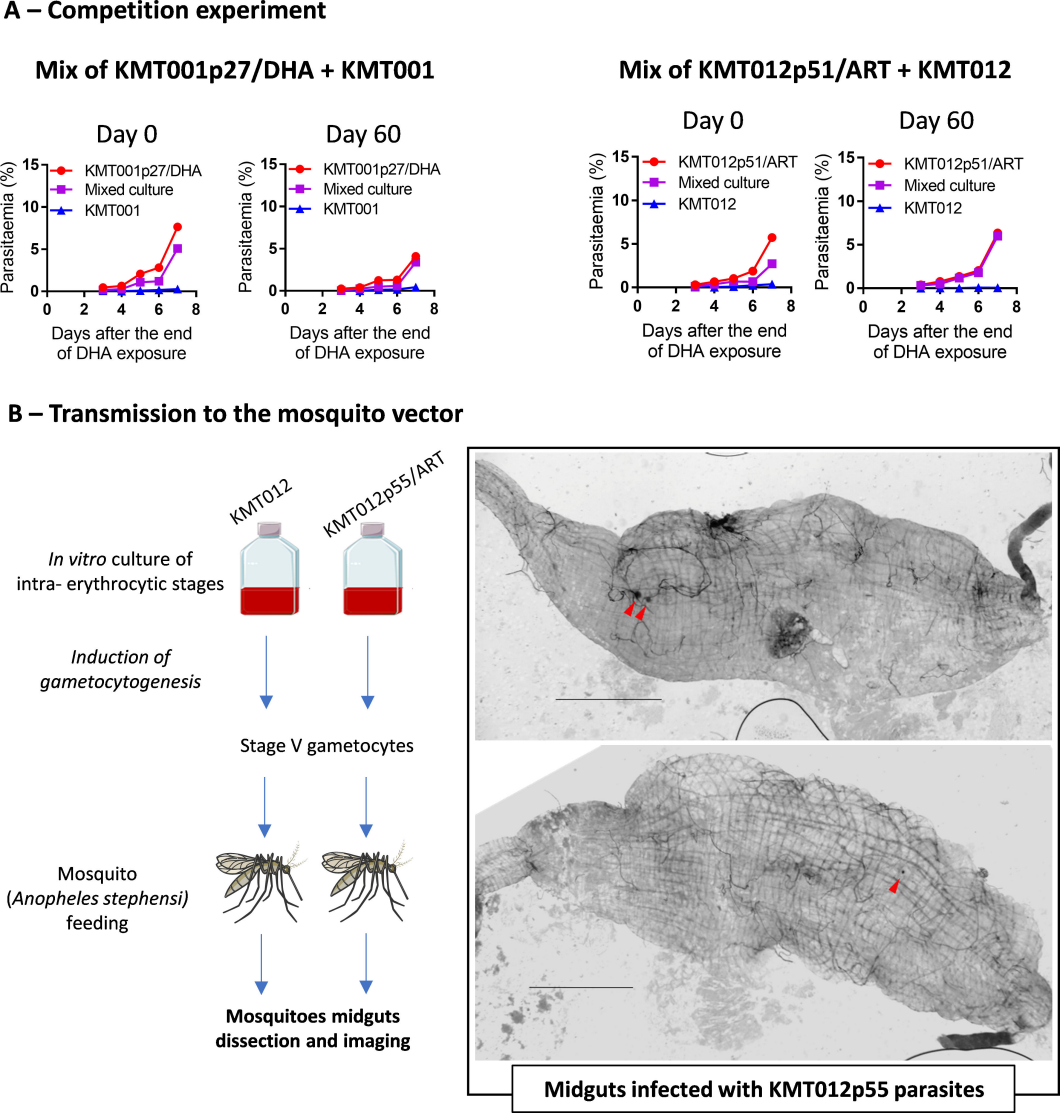
Evaluation of the competitive advantage and transmission abilities of selected parasites. (**A**) *In vitro* evaluation of parasite fitness of selected lines KMT001p27/DHA and KMT012p51/ART against the corresponding ART-susceptible parental lines by evaluation of sensitivity to DHA exposure (700 nM for 6 h) in the RSKA^0–24h^ performed on day 0 and day 60 of drug-free coculture in the separate and mixed cultures of KMT012 and KMT012p51/ART at different times of the competition experiment. (**B**) Evaluation of the transmissibility of KMT012 and KMT012p55 parasites to *Anopheles stephensi* mosquitoes: protocol scheme and imaging of the two midguts infected with KMT012p55 (repeat 1 out of 2, 8 days postinfection). The absence of oocysts in mosquitoes infected with KMT012 gametocytes can be due to other factors of the line than the mutation or trait that was selected. Red arrowheads: oocysts; scale bar: 250 µm.

To assess the competitive advantage of the selected lineages, two coculture experiments were performed with the pairs of lines KMT001p27/DHA - KMT001 and KMT012p51/ART - KMT012 (starting from an initial mixture of equal amounts of resistant and susceptible parasites). On day 0, the RSKA^0–24h^ recrudescence curves of the two mixed cultures showed, as expected, an intermediate profile between the susceptible parental lines and the corresponding selected lines (Fig. 4A). After 2 months of coculture under strictly identical growth and drug-free conditions, the recrudescence curves of the two mixed cultures overlapped with those of the corresponding ART-selected lines, suggesting that the selected parasites had a better competitive advantage than the parental parasites. We then investigated whether these parasites could be transmitted to mosquitoes. Two experiments were performed using the KMT012p55 line and its ART-susceptible parental line KMT012 as a control. Gametocytogenesis was successfully induced in both parasite lines, although very low final gametocytemia was achieved in both inductions (up to 0.4%). Nevertheless, oocysts were observed in two mosquitoes infected with KMT012p55 gametocytes (Fig. 4B), indicating that this line is potentially transmissible to the mosquito vector.

## DISCUSSION

Efficient monitoring of the emergence of ART resistance requires a thorough understanding of its selection process. To this end, we studied *P. falciparum* parasites from different endemic areas that were subjected to *in vitro* ART or DHA pressure. At the end of 3 years of selection experiments, all lineages had acquired the ability to survive DHA exposure, corresponding to the ART resistance phenotype, but only one of them carried a K13 mutation associated with ART resistance (11). A role for the coronin mutations P76S and V424I in ART resistance has been proposed in a study carried out on field isolates from Africa (19). Such a correlation was not evidenced here, as these mutations were carried by several ART-susceptible parental lines. For the coronin mutation P76S, this is consistent with its high prevalence unrelated to ART resistance in many West African countries (25, 26). However, the role of these polymorphisms in the acquisition of ART resistance remains an open question. In-depth genomic studies are in progress to determine the onset of new genomic variations that may be associated with ART resistance in the different selected parasite lines. The regular determination of the ART resistance phenotype of all the parasite lines during the *in vitro* selection process revealed a dynamic process associated with an increasing ability of the parasite population to recover after DHA exposure, with no apparent difference in the speed of resistance acquisition. The ability of parasites to recover after DHA treatment (as evidenced by the RSKA^0–24h^) gradually increased with the number of drug pressure cycles and can also be lost under drug-free conditions. This may reflect the level of fixation of the selected phenotype in the different parasite lineages according to the progress of the selection process. We wondered whether such phenotypic changes might reflect biochemical changes that serve as stepping stones to later irreversible genetic changes, as proposed in the “relay race” model of adaptation (27). This model has previously been proposed in *Plasmodium* in the case of *in vitro* resistance to halofuginone, where an increase in intracellular proline levels preceded the amplification of the gene encoding the cytoplasmic proline tRNA synthetase (28). The observation that acquisition of the *pfk13* P413A mutation in the SMT010/ART lineage, between the 15th and 18th drug pressure cycle (11), was preceded by an increase in parasite recrudescence capacities (Fig. S2H) is consistent with the “relay race” hypothesis. However, formal validation of this hypothesis requires further investigation of parasite developmental and biochemical changes throughout the resistance selection process.

According to the literature, younger parasites are more resistant to ART (9, 29, 30), and the main hypothesis to explain such age dependency is based on the lower hemoglobin uptake occurring in young parasites. Low hemoglobin uptake is maintained for a longer time in parasites carrying a K13 mutation that impairs hemoglobin endocytosis and thereby extended ring stage duration in these K13-mutant parasites; this may lead to reduced ART activation and a greater ability of those parasites to survive ART exposure (30–33). Here, we show that K13-WT ART resistance can be specifically associated with 16 to 24 h old ring parasites without being associated with a prolonged ring stage. This suggests that other unidentified mechanisms may mediate ART resistance and, importantly, warns of the risk of misjudging the emergence and spread of ART resistance when RSA is performed only on 0 to 3 h old rings. In *Plasmodium*, drug resistance is often assumed to confer a fitness cost in the absence of treatment (34). Here, we showed that ART-resistant K13-WT parasites are able to rapidly outgrow ART-susceptible parasites *in vitro* in drug-free conditions. The mechanisms that confer such a selective advantage remain to be identified and may include a faster developmental cycle for the KMT012/ART lineage. Because of their ability to infect mosquitoes, such ART-resistant parasites may easily spread if they emerge in the field.

In conclusion, we show that ART or DHA pressure selects resistant parasites, regardless of their genetic background and geographic origin, which do not necessarily carry classic phenotypic traits and mutations in the *pfk13* gene. This is associated with a progressive increase in the ability of parasite population to survive DHA exposure, which can be detected by novel *in vitro* monitoring assays and may precede the acquisition of an ART resistance-causing mutation. Collectively, these data underline the need to refine current procedures for higher-resolution surveillance of indicators of ART resistance emergence, particularly in Africa.

## SUPPLEMENTAL MATERIAL

Data Set S1 aac.01541-24-s0001.xlsx RSA raw data.

Supplemental material aac.01541-24-s0002.docx Tables S1 to S3; Fig. S1 to S5.

ASM does not own the copyrights to Supplemental Material that may be linked to, or accessed through, an article. The authors have granted ASM a non-exclusive, world-wide license to publish the Supplemental Material files. Please contact the corresponding author directly for reuse.
