# Supplementary material for: Artemisinin pressure in field isolates can select highly resistant Plasmodium falciparum parasites with unconventional phenotype and no K13 mutation
Source: Antimicrob Agents Chemother. 2025 Feb 4;69(3):e01541-24. doi: 10.1128/aac.01541-24 (PMC11881571; doi:10.1128/aac.01541-24)
Supplement: Supplemental material — Tables S1 to S3; Fig. S1 to S5. [file aac.01541-24-s0002.docx]

**Supporting Information for**

**Artemisinin pressure in field isolates can select highly resistant *Plasmodium falciparum* parasites with unconventional phenotype and no K13 mutation**

Lucie Paloque, Luana Mathieu, Marion Laurent, Romain Coppée, Stéphanie Blandin, Pascal Campagne, Jean-Michel Augereau, Lise Musset, Françoise Benoit-Vical

Address for correspondence : Lucie Paloque, Laboratoire de chimie de coordination, 205 route de Narbonne, 31077 Toulouse, France ; email : [lucie.paloque@lcc-toulouse.fr](mailto:lucie.paloque@lcc-toulouse.fr)

**This file includes:**

Table S1 to S3

Figure S1 to S5

**Table S1**: For strains under artemisinin pressure, detail of concentrations used for each cycle of drug pressure.

| **Drug pressure cycle** | **Artemisinin concentration (nM)** | **Drug pressure cycle** | **Artemisinin concentration (nM)** |
| --- | --- | --- | --- |
| **1** | **10** | **21 - 23** | **1400** |
| **2** | **18** | **24 - 26** | **1700** |
| **3** | **26** | **27 - 29** | **2400** |
| **4** | **35** | **30 – 32** | **2600** |
| **5 - 6** | **53** | **33 - 35** | **3500** |
| **7 - 8** | **106** | **36 – 38** | **4400** |
| **9 - 10** | **200** | **39 – 41** | **5300** |
| **11 - 13** | **300** | **42 – 44** | **7000** |
| **14 - 15** | **500** | **45 – 47** | **8800** |
| **16 - 17** | **750** | **48 – 50** | **13000** |
| **18 - 20** | **1000** | **51 - 55** | **15000** |

**Table S2**: List of primer sets used for PCR and Sanger sequencing

| **Gene name** | **Gene ID** | **PCR primer sets (5’-3’)** |
| --- | --- | --- |
| *pfk13** | PF3D7_1343700 | Fw – GGGAATCTGGTGGTAACAGC  Rev- CGGAGTGACCAAATCTGGGA |
| *pfcoronin* | PF3D7_1251200 | Fw – ATGTATAATGTTCCTTTAATCAAGA  Rev – CTTTAAACTCCATAATTTCAATTCTC |
|  |  | Fw – AAGTTCTTTACAATGGGATATCG  Rev – TACCGTTGCTGTACTTTTACAC |
| **Gene name** | **Gene ID** | **Sanger sequencing primers (5’-3’)** |
| *pfk13** | PF3D7_1343700 | Fw – GGGAATCTGGTGGTAACAGC |
|  |  | Fw–GCCTTGTTGAAAGAAGCAGA |
|  |  | Rev-CGCCAGCATTGTTGACTAAT |
|  |  | Rev- CGGAGTGACCAAATCTGGGA |
| *pfcoronin* | PF3D7_1251200 | Fw – ATGTATAATGTTCCTTTAATCAAGA |
|  |  | Rev – CTTTAAACTCCATAATTTCAATTCTC |
|  |  | Fw – AAGTTCTTTACAATGGGATATCG |
|  |  | Rev – TACCGTTGCTGTACTTTTACAC |

** The pfk13 primer sets were previously published in (*[*https://www.wwarn.org/tools-resources/procedures/pcr-and-sequencing-genotyping-candidate-plasmodium-falciparum-artemisinin*](https://www.wwarn.org/tools-resources/procedures/pcr-and-sequencing-genotyping-candidate-plasmodium-falciparum-artemisinin)*).*

**Table S3:** Genotyping of the *pfk13* and *pfcoronin* genes, done in the *P. falciparum* parental lines used in this study, and in the selected lines annotated pX (“p” for pressure, “X” for the number of pressure cycles and ART/DHA for the drug used for pressure). WT: wild type.

| **Strains** | ***pfk13* (PF3D7_1343700)** | ***pfcoronin***  **(PF3D7_1251200)** |
| --- | --- | --- |
| **KMT001** | K189T* | WT |
| KMT001p15/ART | K189T* | nd |
| KMT001p22/ART | K189T* | WT |
| KMT001p30/ART | K189T* | nd |
| KMT001p14/DHA | K189T* | nd |
| KMT001p25/DHA | K189T* | WT |
| KMT001p35/DHA | K189T* | nd |
| **KMT004** | K189T* | S183G* |
| KMT004p15/ART | K189T* | nd |
| KMT004p18/ART | K189T* | nd |
| KMT004p21/ART | K189T* | nd |
| KMT004p28/ART | K189T* | nd |
| KMT004p36/ART | K189T* | S183G* |
| KMT004p44/ART | K189T* | nd |
| **KMT012** | K189N* | P76S*, S183G*, V424I* |
| KMT012p21/ART | K189N* | nd |
| KMT012p24/ART | K189N* | nd |
| KMT012p34/ART | K189N* | P76S*, S183G*, V424I* |
| KMT012p44/ART | K189N* | nd |
| KMT012p54/ART | K189N* | nd |
| KMT012p55/ART | K189N* | nd |
| **KMT102** | K189T* | S183G* |
| KMT102p24/ART | K189T* | nd |
| KMT102p32/ART | K189T* | nd |
| KMT102p41/ART | K189T* | S183G* |
| KMT102p49/ART | K189T* | nd |
| **SMT010** | WT | WT |
| SMT010p15/ART | WT | nd |
| SMT010p18/ART | **P413A^#^** | nd |
| SMT010p21/ART | **P413A^#^** | nd |
| SMT010p24/ART | **P413A^#^** | nd |
| SMT010p10/DHA | WT | nd |
| SMT010p17/DHA | WT | nd |
| SMT010p27/DHA | WT | WT |
| SMT010p36/DHA | WT | nd |
| **S691** | WT | V424I*, E519K* |
| S691p40/DHA | WT | V424I*, E519K* |
| **U236** | K189T* | V424I*, E519K* |
| U236p40/DHA | K189T* | V424I*, E519K* |
| **O141-A** | WT | V424I*, E519K* |
| O141-Ap40/DHA | WT | V424I*, E519K* |
| **IPC5188** | WT | WT |
| IPC5188p40/DHA | WT | WT |

** SNPs never associated with artemisinin resistance*

*^#^ Previously published in Paloque 2022 AAC Jan 18;66(1): e0132021*

*nd: not dertermined*

**Figure S1**

**
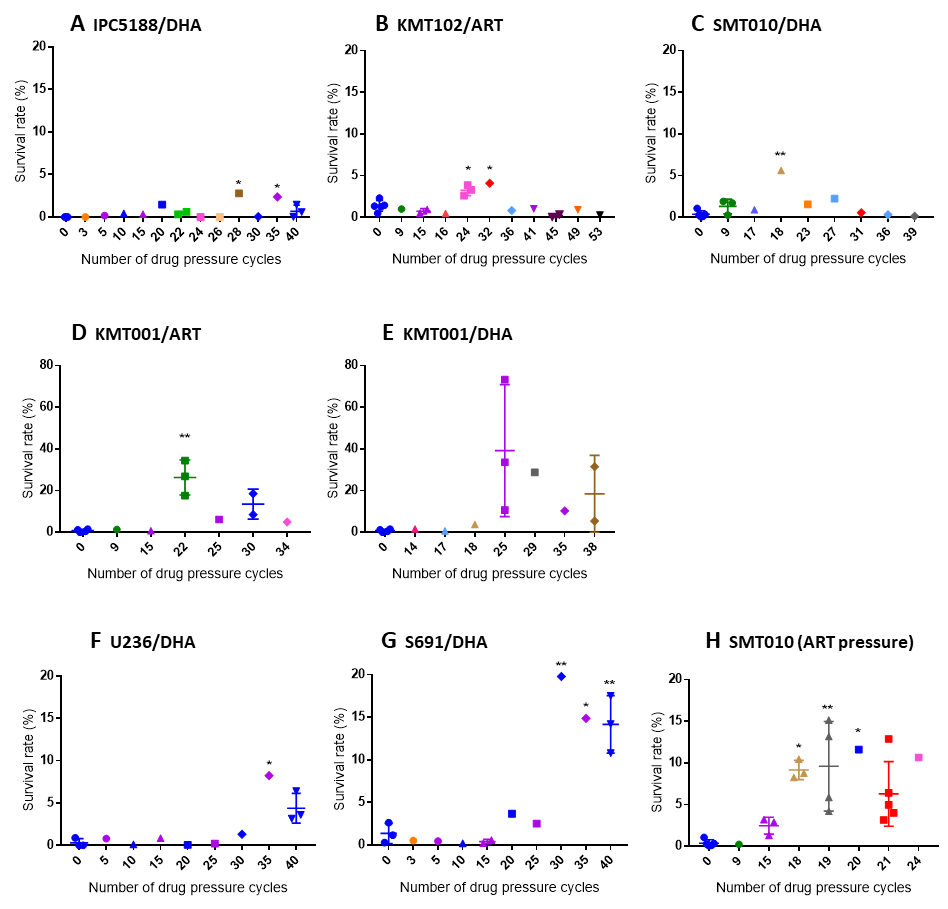
**

**Figure S1: *In vitro* evaluation of the susceptibility of parasite lines throughout the drug resistance selection process in the RSA^0-3h^**. **(A-H)** RSA^0-3h^ mean survival rate (± SEM) of parasites exposed to 700 nM DHA for 6 h compared to the control condition for each lineage at different times of the selection process (after X drug cycles). Statistical significance (one-way ANOVA Dunnett’s *t* test) was determined for each pressure cycle compared to p0. *(*p-*value < 0.05); **(*p*-value < 0.01). IPC5188 originates from Cambodia; KMT102, SMT010, and KMT001 originate from Mali; U236 and S691 originate from French Guiana.

**Figure S2**

**
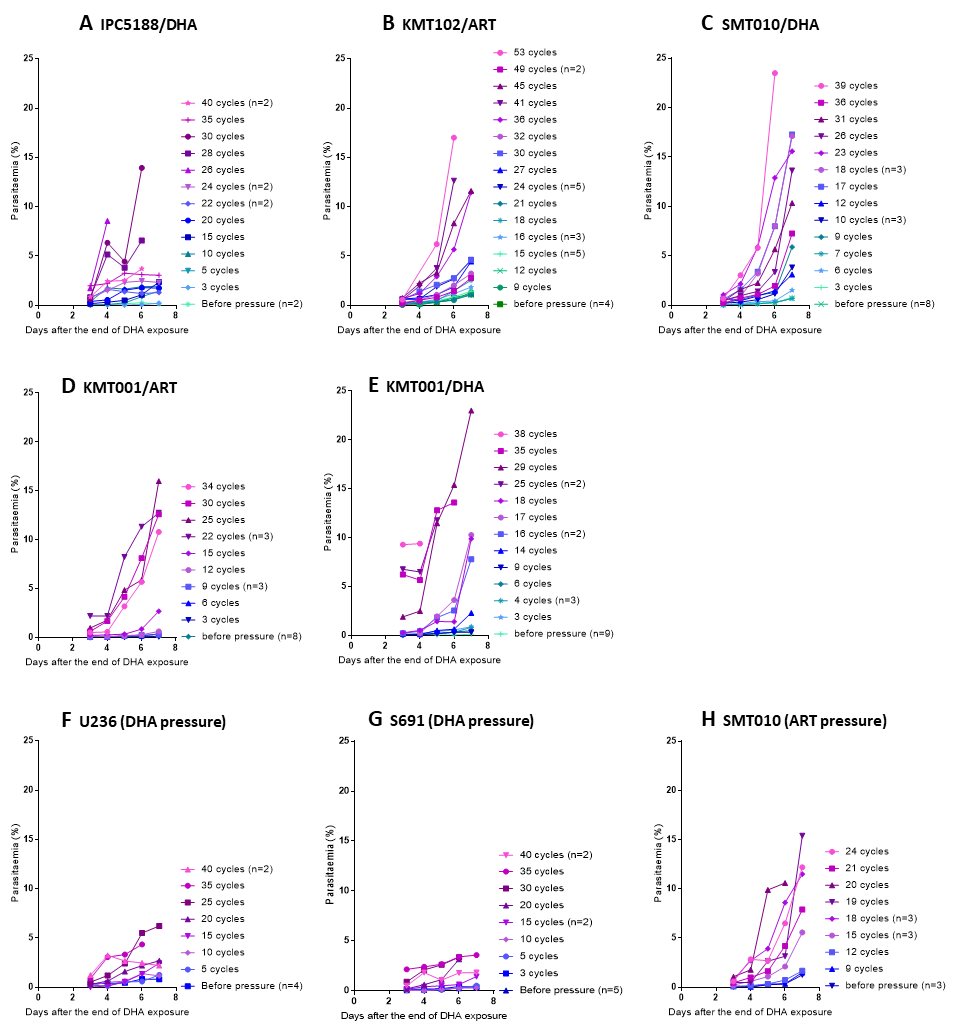
**

**Figure S2: *In vitro* evaluation of the susceptibility of parasite lines throughout the drug resistance selection process in the RSKA^0-24h^**. **(A-H**) Recrudescence curves of parasites over seven days (parasitemia as a function of time) after the end of DHA exposure for each lineage at different times of the selection process (after X drug cycles). *Some curves end earlier because high parasitemia leads to impaired ability of parasites to reinvade red blood cells*.

**
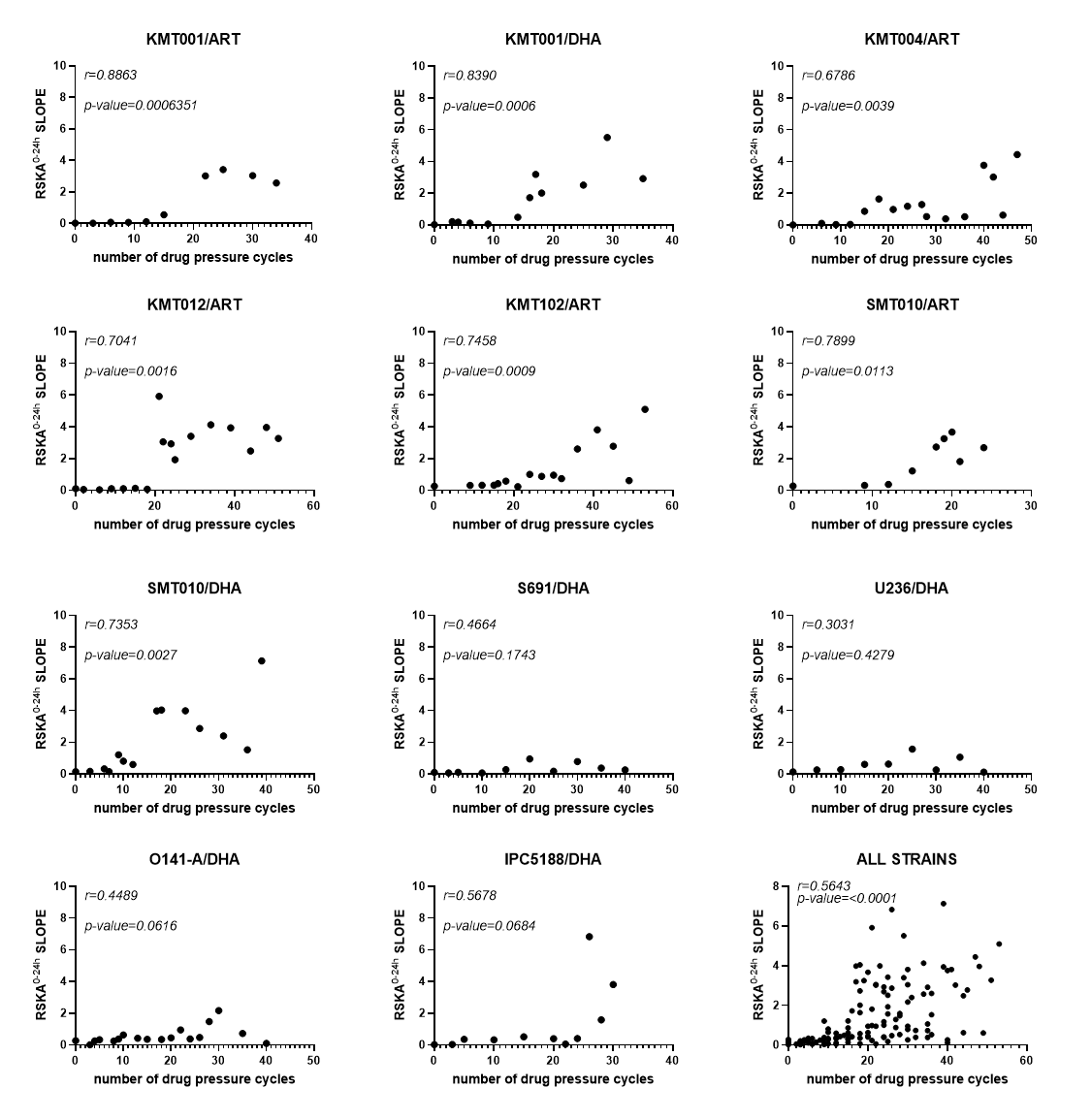
**

**Figure S3: *In vitro* evaluation of the susceptibility of parasite lines throughout the drug resistance selection process in the RSKA^0-24h^**. Correlation between RSKA^0-24h^ SLOPE and number of drug pressure cycles for all selected lineages (both ART and DHA pressures). The SLOPE of each recrudescence curves of parasites over seven days after the end of DHA exposure was calculated using Excel Software.

If the analysis of RSKA^0-24h^ data is restricted to SLOPE determination, important pieces of information will be missing: the parasitaemia at D3, and the ability of parasites to proliferate at medium to high level of parasitemia in recrudescence experiments.

**Figure S4**


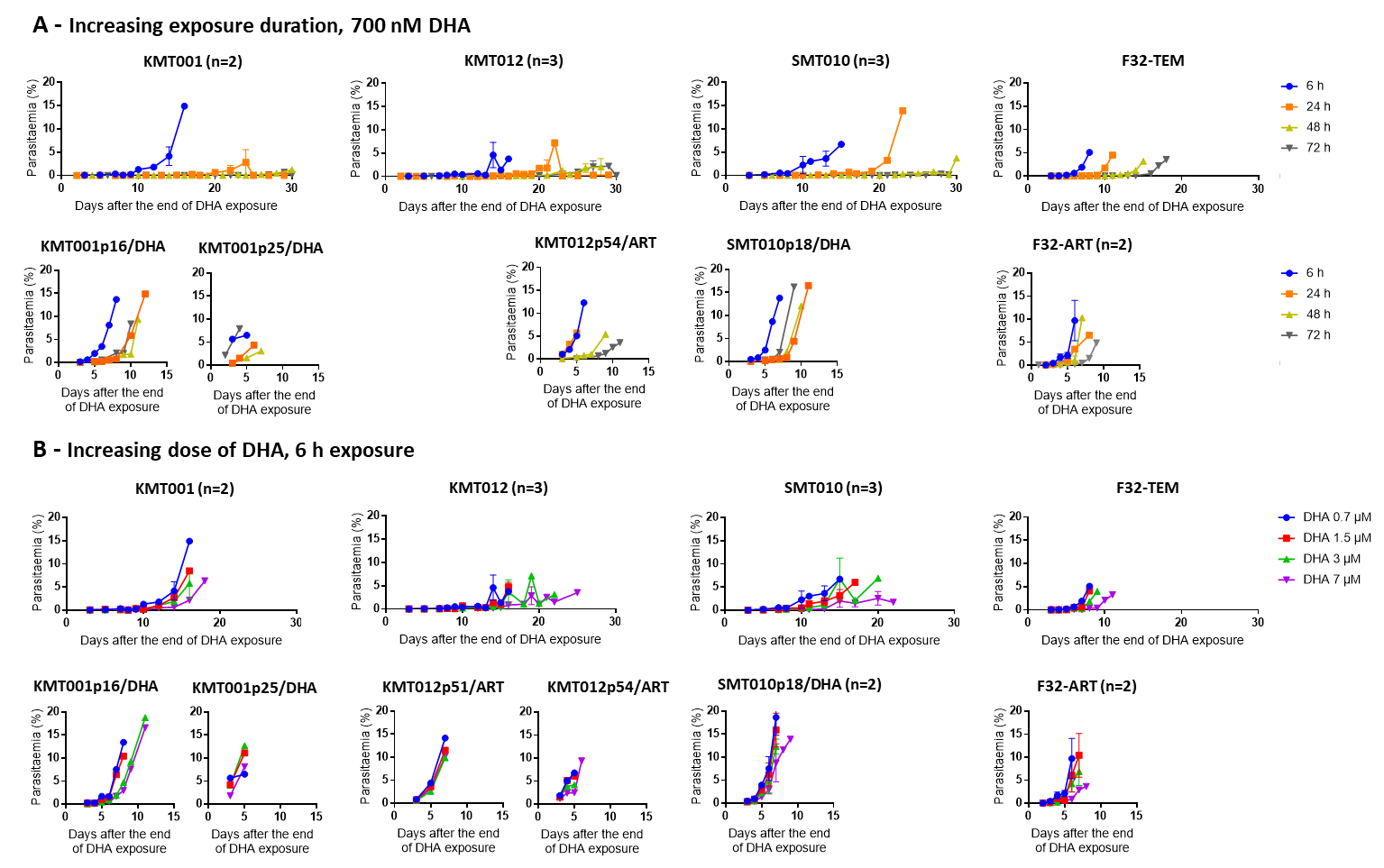


**Figure S4** : *In vitro* evaluation of parasite sensitivity to DHA exposure in the RSKA according to: **(A)** increasing DHA exposure duration (700 nM for 6 h to 72 h) or **(B)** increasing DHA doses (from 0.7 to 7 µM for 6 h). The experiments were carried out on 0-24 h old rings, except for the line KMT012p54/ART (16-20 h old rings according to previous results showing a higher ability to withstand DHA exposure at old-ring stage for this line). No differences were observed between these two conditions for the KMT012/ART lineage as sorbitol synchronized parasites often contained a majority of middle to late ring-stage.

We found that increasing the duration of DHA exposure (from 6 h to 72 h) prevented or delayed the recovery of ART-susceptible parasites but not that of ART-selected lines. Similarly, selected parasite lines retained a high recovery capacity even when DHA doses were increased, highlighting again their elevated ART resistance.

**Figure S5**


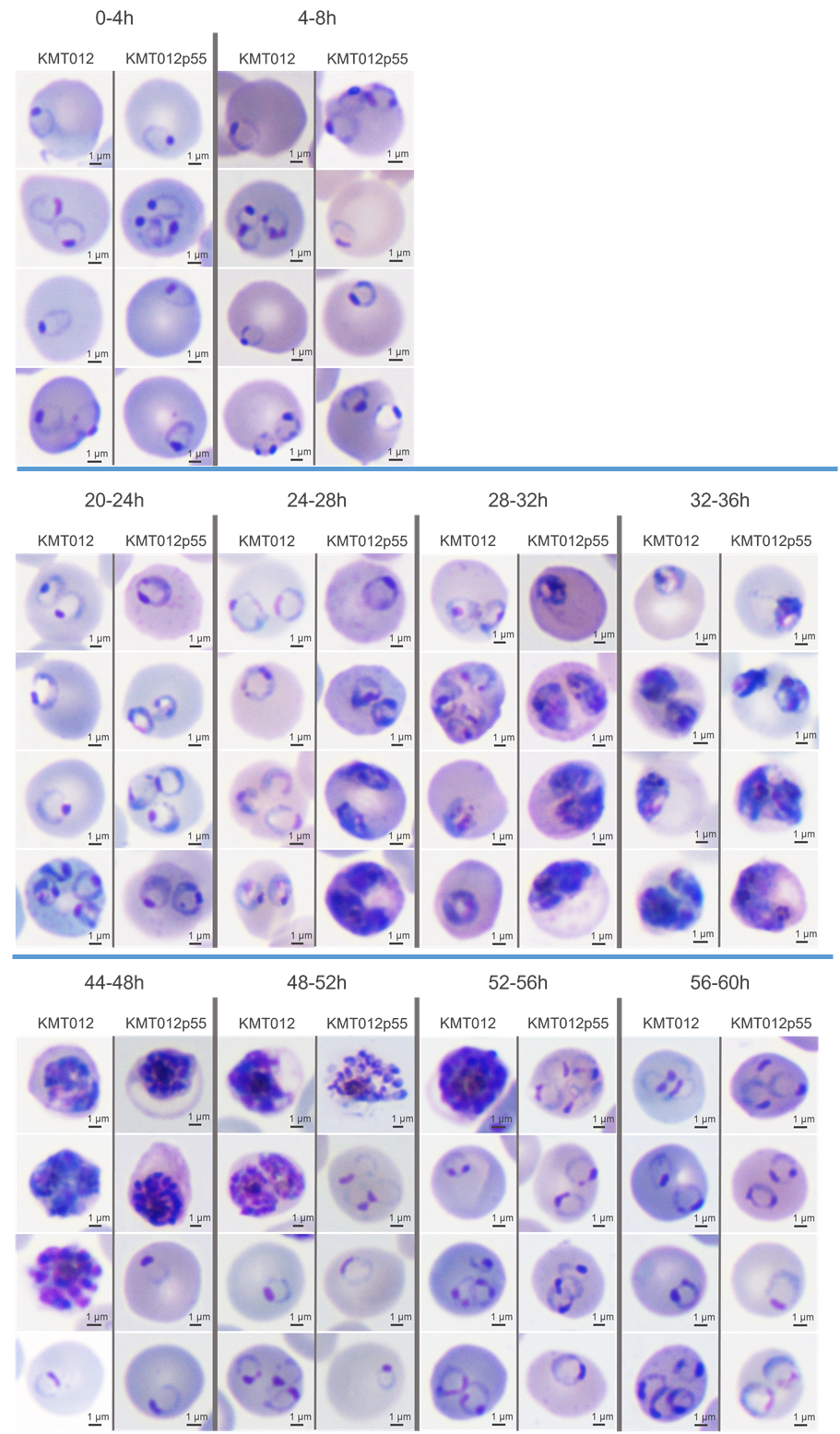


**Figure S5:** Morphological analysis of the intraerythrocytic development cycle for KMT012 and KMT012p55/ART lines by microscopic examination (DiffQuick-stained blood smear, magnification 100 with immersion oil, scale bar: 1 µm). Parasite cultures were tightly synchronised at 0-4 h post-invasion (by Percoll-Sorbitol treatment) and blood smears were taken at 4, 8, 24, 28, 32, 36, 48, 52, 56 and 60 h.
